# Adapting the German demand planning guideline for physiotherapy: a cross-sectional analysis from Lower Saxony

**DOI:** 10.1007/s43999-025-00081-1

**Published:** 2025-12-12

**Authors:** Anne Griese, Frank Schüssler

**Affiliations:** 1 Institute of Midwifery, Charité - Universitätsmedizin, Berlin, Germany; 2 Institute for Applied Photogrammetry and Geoinformatics, Jade University of Applied Sciences, Oldenburg, Germany

**Keywords:** Physiotherapy, Healthcare planning, Supply and demand, Health geography, Workforce distribution

## Abstract

**Background:**

The Demand Planning Guideline aims to ensure equitable and needs-based outpatient care in Germany but currently excludes allied health professions such as physiotherapy. This study investigates current and projected regional supply levels of physiotherapists in Lower Saxony, a federal state in Germany, applying the physician-based planning methodology to highlight workforce imbalances.

**Methods:**

This cross-sectional study used administrative address data from January 2021 to determine the number and distribution of physiotherapists. These data were geolinked at the district level and combined with population projections for 2040 from the Federal Institute for Research on Building, Urban Affairs and Spatial Development (BBSR). The methodology of the Demand Planning Guideline was applied, including age-adjusted correction factors, to assess regional care levels and future trends.

**Results:**

Significant regional variation in supply was found. In 2020, no district was formally undersupplied, but corrected supply levels ranged from 55.7% to 146.4%. By 2040, assuming a constant workforce, disparities are expected to increase, with 17 districts becoming oversupplied and none formally falling below the 50% threshold, despite indications of structural undersupply in several areas.

**Conclusion:**

Adapting the Demand Planning Guideline to include physiotherapy enables the identification of regional supply disparities and better forecasts of future care needs. A more inclusive planning framework is required to ensure equitable access and sustainable care provision in light of demographic change.

**Supplementary information:**

The online version contains supplementary material available at 10.1007/s43999-025-00081-1.

## Introduction

Demand planning in healthcare aims to ensure that the provision of medical services meets population needs while avoiding over- and undersupply [1]. It was introduced in many countries to promote equitable access and optimize resource allocation [2,3]. Germany introduced demand planning formally in 1977 as part of the Act on the Further Development of Health Insurance Fund Law [4].

In Germany, demand planning is currently restricted to outpatient physician care, while hospital services are planned at the federal state level through state-specific hospital laws [5]. In other countries, including the Netherlands and Norway, broader frameworks have been implemented that also integrate allied health professions [6,7]. The UK National Health Service (NHS) conducts regional health needs assessments to inform workforce deployment [8]. These models share the goal of aligning workforce availability with demographic and regional indicators to reduce disparities and ensure continuity of care [9].

Germany’s narrow scope excludes key allied health professions such as physiotherapists, who provide a large share of outpatient treatment. In 2020, over 40 million therapeutic prescriptions resulted in 302 million treatment sessions, with physiotherapy accounting for 83.4% of all sessions [10]. Utilization increases significantly with age and peaks among individuals aged 80–84. At the same time, the number of new physiotherapy graduates is declining, and the profession is classified as a bottleneck occupation by the Federal Employment Agency [11]. Despite clear indications of a growing imbalance between supply and demand, Germany lacks a national registry for physiotherapists, and planning efforts rely on proxy indicators such as billing data.

International evidence suggests that structured health workforce planning across multiple professions can improve care quality and reduce general practitioners’ workload [12,13]. Against this background, the present study investigates whether existing physician-based planning logic can be applied to physiotherapy to identify regional disparities in care.

## Demand planning guideline

Germany’s Demand Planning Guideline defines how adequate physician supply is determined and how planning areas are classified. The system was initially based on historical provider-to-population ratios that were assumed to represent sufficient levels of care. Over time, these benchmarks were refined into modified ratios that incorporate demographic and epidemiological indicators such as age, gender, and morbidity [4]. Within this framework, a supply level (SL) is calculated for each planning area as the ratio of actual to target provision, with 100% representing adequate supply. Thresholds guide the allocation of new providers: for example, undersupply is defined as below 75% in general practice or below 50% in specialist care, while regions above 110% are classified as adequately supplied [14]. To reflect population needs more accurately, ratios are adjusted for demographic structure through age-specific utilization factors. In addition, the framework incorporates spatial planning principles to account for interdependencies between urban centers and surrounding rural districts. Larger cities often serve as supply hubs, while peripheral districts may rely on nearby centers for service provision [15,16]. By integrating demographic adjustment and spatial differentiation, the guideline enables a systematic and needs-oriented distribution of providers across regions. While it has thus far been applied exclusively to outpatient physicians, its structured methodology provides a transferable model that can also be used to assess other health professions.

The objective of this cross-sectional study is to assess the current regional supply of physiotherapists in Lower Saxony, a federal state in Germany, and to identify disparities using the methodology of the physician-based Demand Planning Guideline. It also projects regional supply levels for the year 2040 based on demographic change. The study hypothesizes that formal application of the guideline will reveal spatial mismatches that remain unaddressed in current planning efforts.

## Methods

The study was conducted and reported in accordance with the Strengthening the Reporting of Observational Studies in Epidemiology (STROBE) guideline [17].

### Study design and setting

This cross-sectional study was conducted in Lower Saxony, a federal state in northwestern Germany comprising 45 districts and independent cities.

### Data sources and participants

All population data were obtained from the 2021 population projection of the Federal Institute for Research on Building, Urban Affairs and Spatial Development (BBSR) [18]. According to this projection, Lower Saxony had 7,958,600 inhabitants in 2020, and the mean age of the population was 44.8 years. For the year 2040, the BBSR forecasts a slight decline in the state’s population to 7,787,500 inhabitants, with the mean age increasing to 46.3 years.

Nexiga compiles its datasets from multiple verified sources, including official statistics from state and federal statistical offices, proprietary market research, and validated commercial address records (e.g., Deutsche Telekom, Deutsche Post), which ensures a high level of accuracy and reliability.

Data on physiotherapy facilities were derived from administrative business address records provided by Nexiga GmbH. The unit of analysis was the facility, defined as each unique business address offering physiotherapy services. The dataset includes both individual providers registered under their name and group practices or companies. Nexiga compiles its datasets from official statistics, proprietary market research, and validated commercial address records (e.g., Deutsche Telekom, Deutsche Post), ensuring high accuracy and reliability. A cleaning process was applied to remove duplicates, addresses outside of Lower Saxony, and entries that could not be geocoded. The final dataset thus represents all unique and valid physiotherapy facilities in the state as of January 1, 2021. Geospatial linkage and mapping of these data were conducted using QGIS (version 3.22).

### Variables and measurement

The primary outcome was the district-level supply of physiotherapists, measured as the number of facilities per 100,000 inhabitants. In the absence of an official target ratio for physiotherapists, the 2020 statewide average (4,483.7 inhabitants per facility) was used as a benchmark, following the logic of the physician-based planning guideline [4]. To account for demographic variation, the supply level was adjusted using age-specific utilization rates of physiotherapy services derived from national billing data reported by the Scientific Institute of the AOK (WIdO) [10]. In 2020, 261 per 1,000 insured persons aged 65 years and older used physiotherapy services, compared to 126 per 1,000 among those younger than 65 years. Based on these values, a service requirement multiplier of 2.1 was calculated, indicating that people aged 65 years and older have more than twice the physiotherapy demand of younger population groups. The adjusted supply level was then expressed as a percentage of the statewide benchmark.

### Statistical analysis and classification

Descriptive statistics were used to assess regional variation in both actual and adjusted supply levels. Districts were classified using two methods: (1) according to the Demand Planning Guideline thresholds ( < 50%, 50–110%, > 110%) and (2) into quintiles to enable more detailed comparisons across the full distribution. Projections for 2040 were generated under the assumption of a constant number of physiotherapists.

### Bias and limitations

Efforts to reduce bias included data cleaning and geocoding validation. However, the dataset lacked information on part-time employment, caseload, and morbidity, potentially leading to overestimation of actual service capacity. Moreover, the projection assumes a static workforce, which may underestimate future shortages given demographic trends and training constraints.

### Study size

This was a full-population analysis; no sampling procedures were applied. All physiotherapists listed in the administrative dataset for Lower Saxony were included.

## Results

### Study population and data quality

After data cleaning and geocoding, a total of 1,775 physiotherapy facilities across the 45 districts and independent cities of Lower Saxony were included in the analysis. All entries were successfully geolocated and linked to district-level population data. No missing data were present for the main variables.

### Geographic distribution

**Fig. 1:**
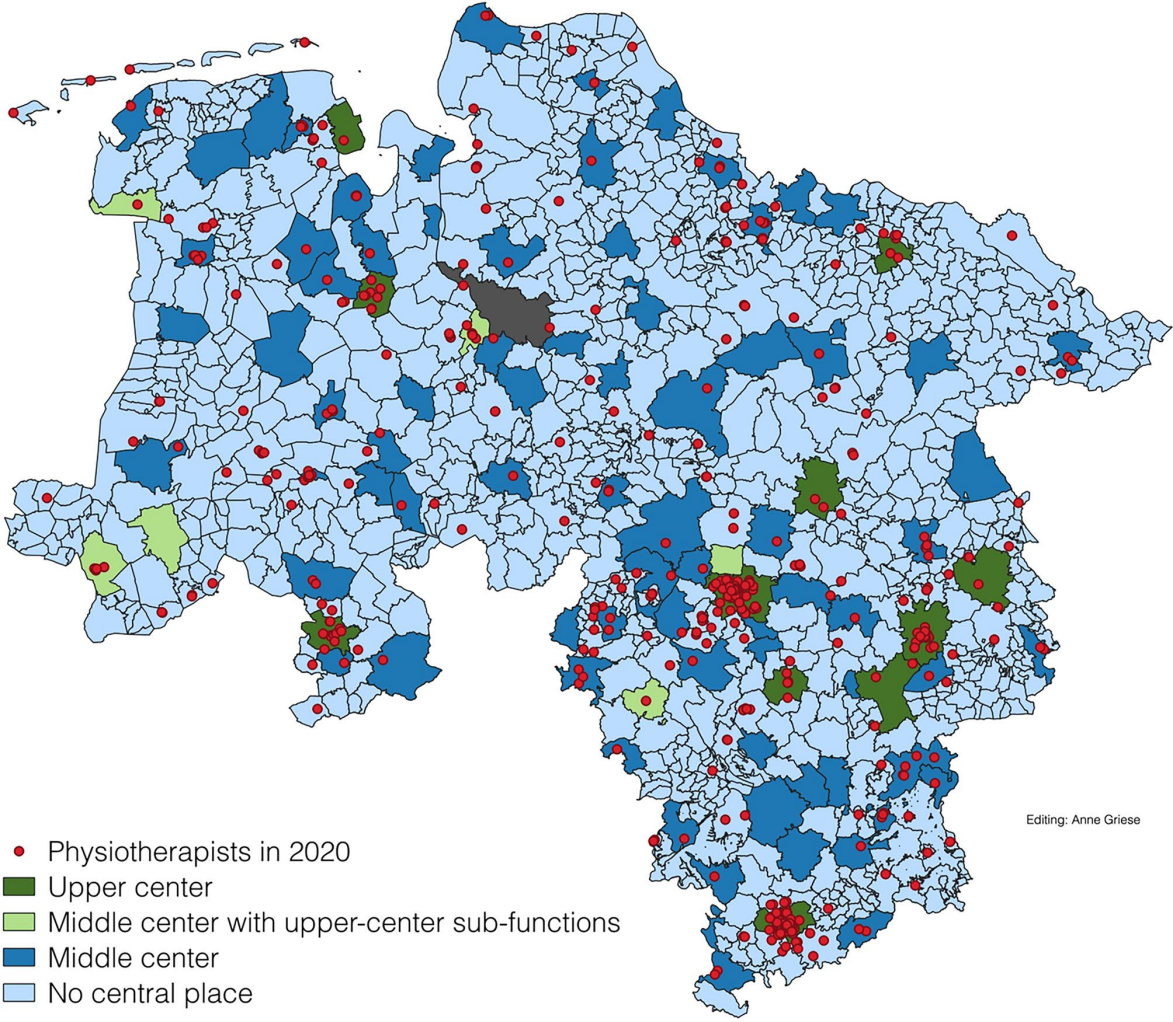
Distribution of physiotherapists in Lower Saxony at district level considering central places (Data sources: nexiga GmbH (2021); BBSR (2021); OpenGeoData.NI. (2021))

Figure 1 shows the geographic distribution of physiotherapists across Lower Saxony. Urban centers such as the Hanover region and Braunschweig exhibited higher facility densities, while rural and peripheral areas, including Wolfsburg and Lüchow-Dannenberg, showed significantly lower concentrations.

### Descriptive statistics and benchmark calculation

The benchmark supply ratio was calculated at 4,483.7 inhabitants per physiotherapist, based on the total state population of 7,958,600 and the number of included facilites (*n* = 1,775). Regional supply levels showed substantial variation:

- The lowest supply was observed in Wolfsburg (7,825 inhabitants per facility), Stade, and Leer.
- The highest supply was recorded in Hameln-Pyrmont (2,924), Göttingen, and Lüchow-Dannenberg.

### Demographically adjusted supply levels (2020)

**Fig. 2:**
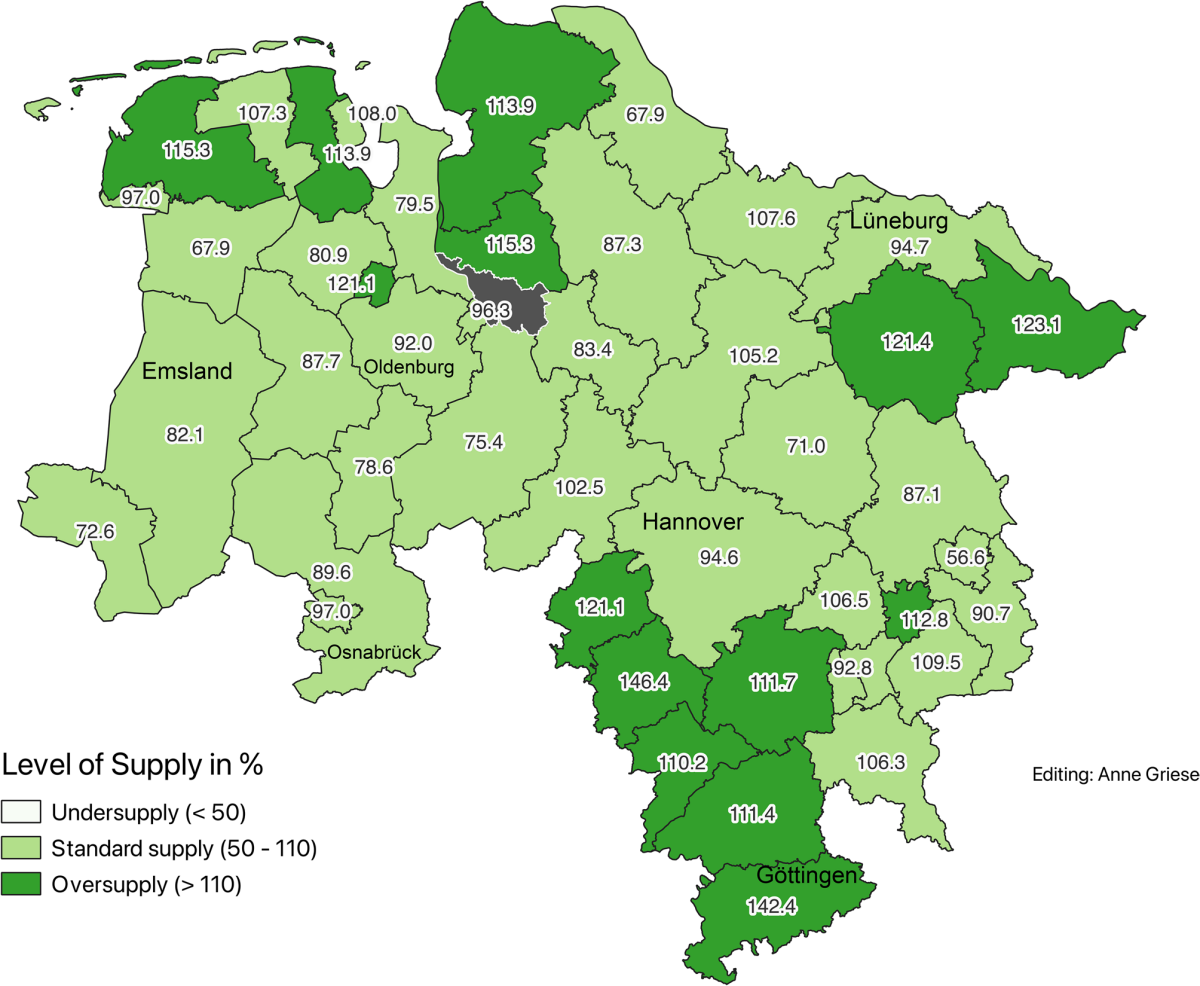
Corrected supply levels according to demand planning guideline in 2020 (Data sources: nexiga GmbH (2021); OpenGeoData.NI. (2021))

**Fig. 3:**
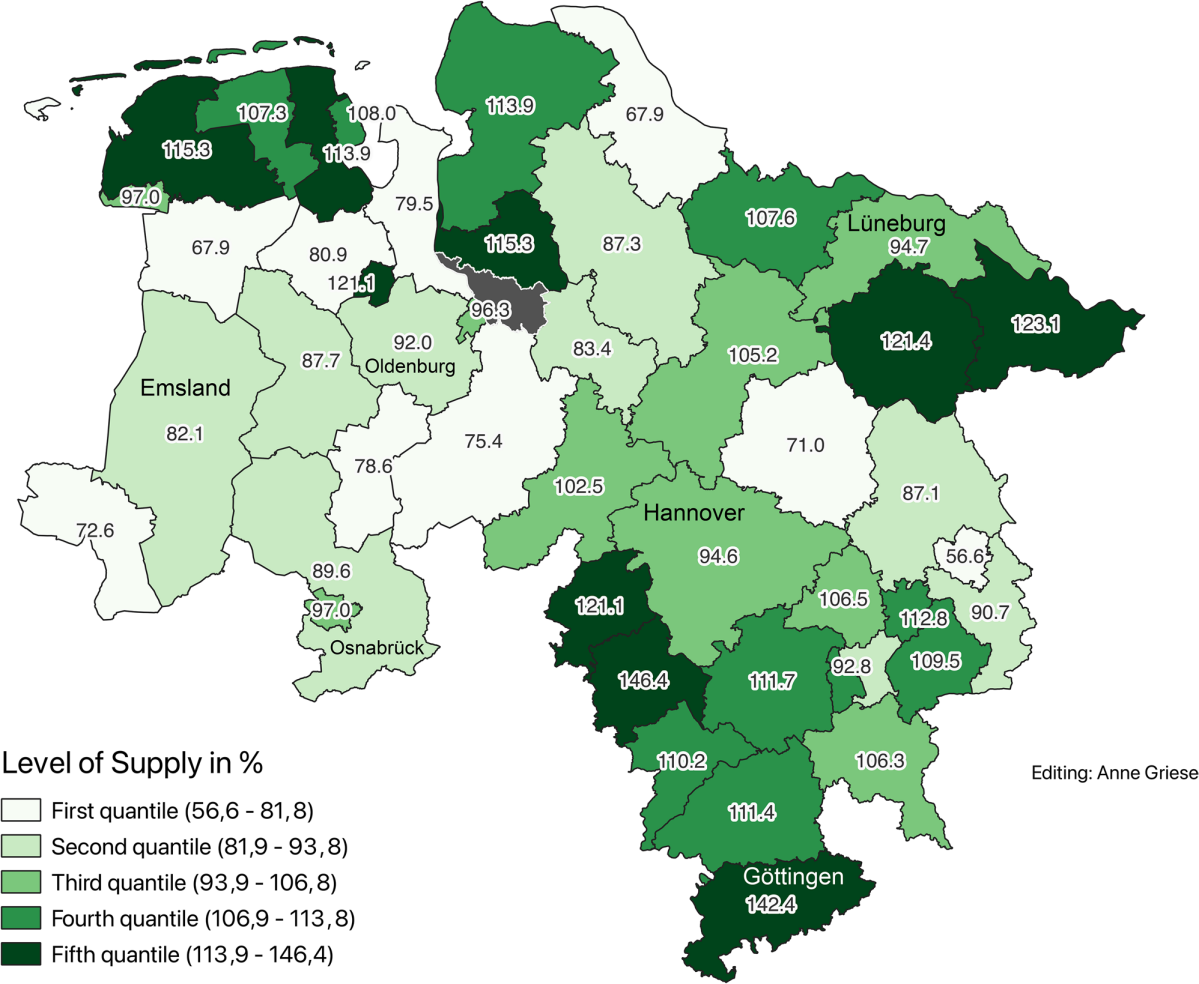
Corrected supply levels by quantiles in 2020 (Data sources: nexiga GmbH (2021); OpenGeoData.NI. (2021))

To account for differences in age structure, a service multiplier of 2.1 was applied to the population aged 65 and older. This adjustment revealed corrected supply levels ranging from 55.7% (Wolfsburg) to 146.4% (Hameln-Pyrmont). Districts were classified using two methods: (Figs. 2 and 3)

Planning guideline classification:

- Undersupply ( < 50%): none
- Adequate supply (50–110%): 31 districts
- Oversupply ( > 110%): 14 districts

Quintile-based classification for finer comparison:

- First quintile (lowest supply): 56.5%–80.9%
- Fifth quintile (highest supply): 113.9%–146.4%

To provide a concise overview, Table 1 summarizes population size, physiotherapy facilities, supply ratios, and corrected supply levels across all districts in 2020 and projected for 2040, including the proportion of residents aged 65 years and older. This table highlights the substantial variation across districts, while detailed values are available in Supplementary Tables S1 and S2.

**Table 1:** Summary of regional variation in Lower Saxony (2020 and 2040)

Indicator	Min	Median	Max
Population	47,700	137,600	1,160,800
Physiotherapy facilities (2020)	11	31	246
Corrected supply level (2020)	56.60	97.00	146.40
Corrected supply level (2040)	55.7	96.2	151.5
Actual Supply Ratio (2020)	2924.0	4594.1	7825.0
Population Aged 65+ (2020) in %	16.8	22.8	28.5
Population Aged 65+ (2040) in %	22.2	29.1	34.2

### Projections for 2040

**Fig. 4:**
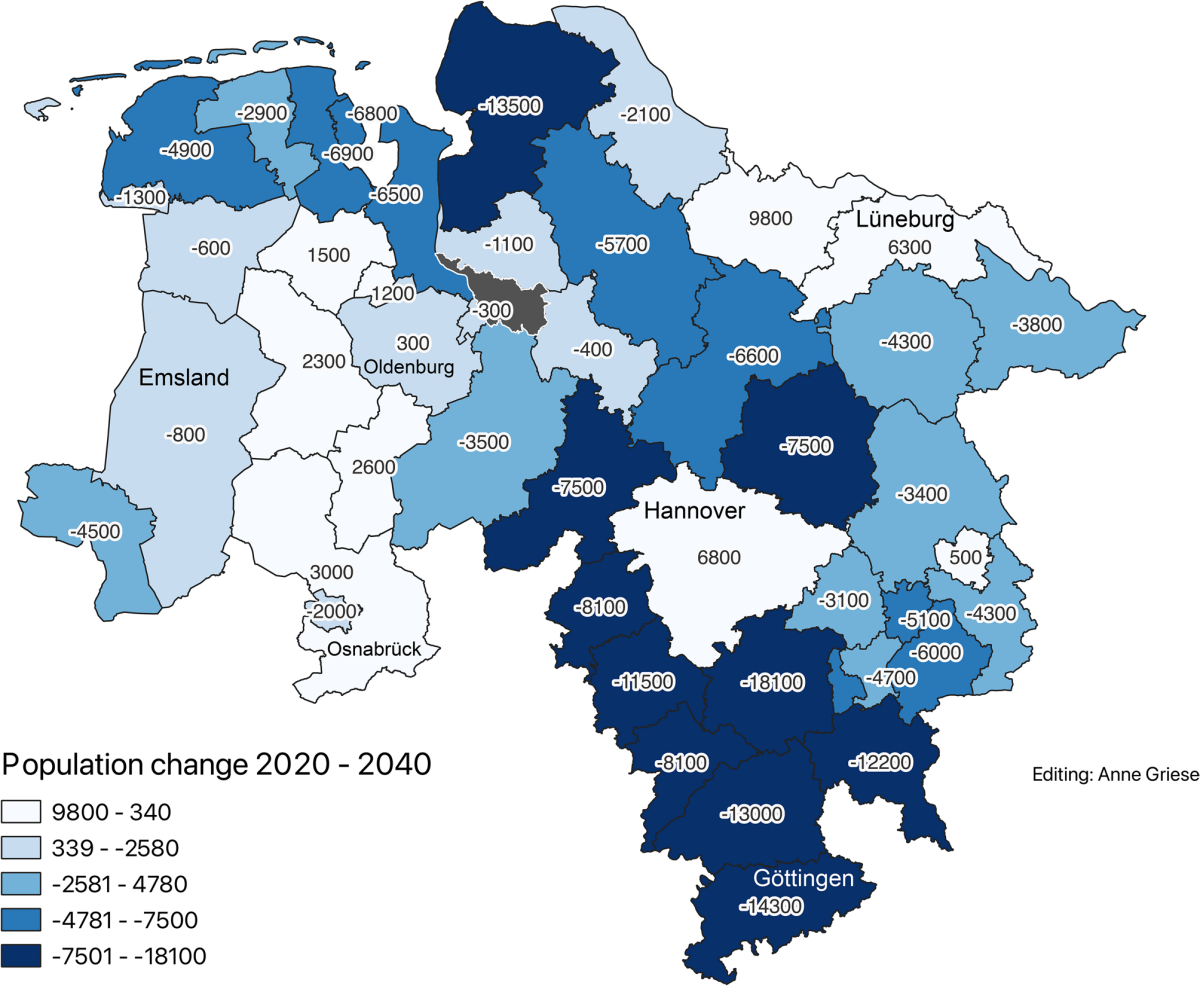
Projected population change from 2020 to 2040 (Data sources: BBSR (2021); OpenGeoData.NI. (2021))

**Fig. 5:**
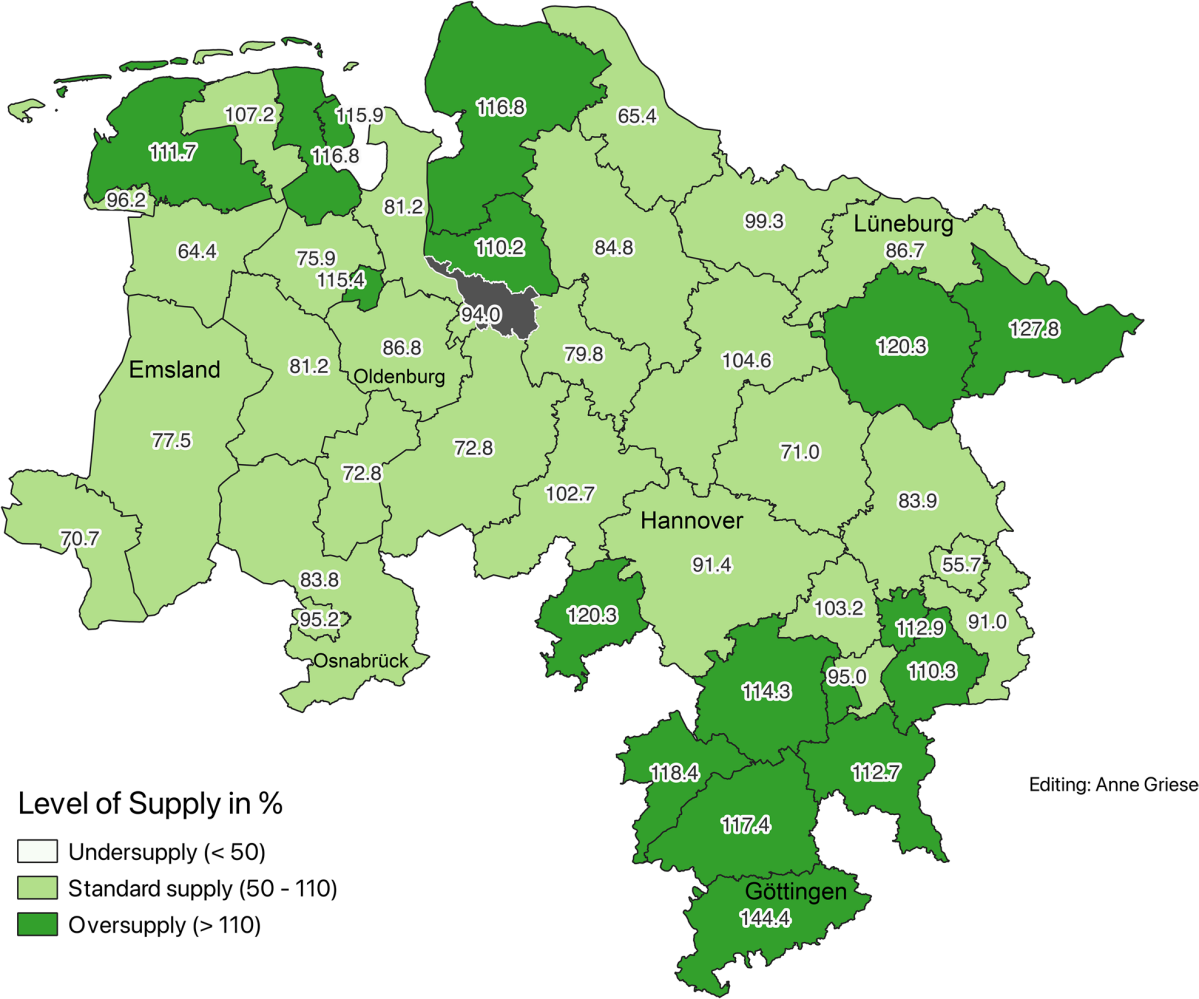
Corrected supply levels according to demand planning guideline in 2040 (Data sources: BBSR (2021); Nexiga GmbH (2021); OpenGeoData.NI. (2021))

**Fig. 6:**
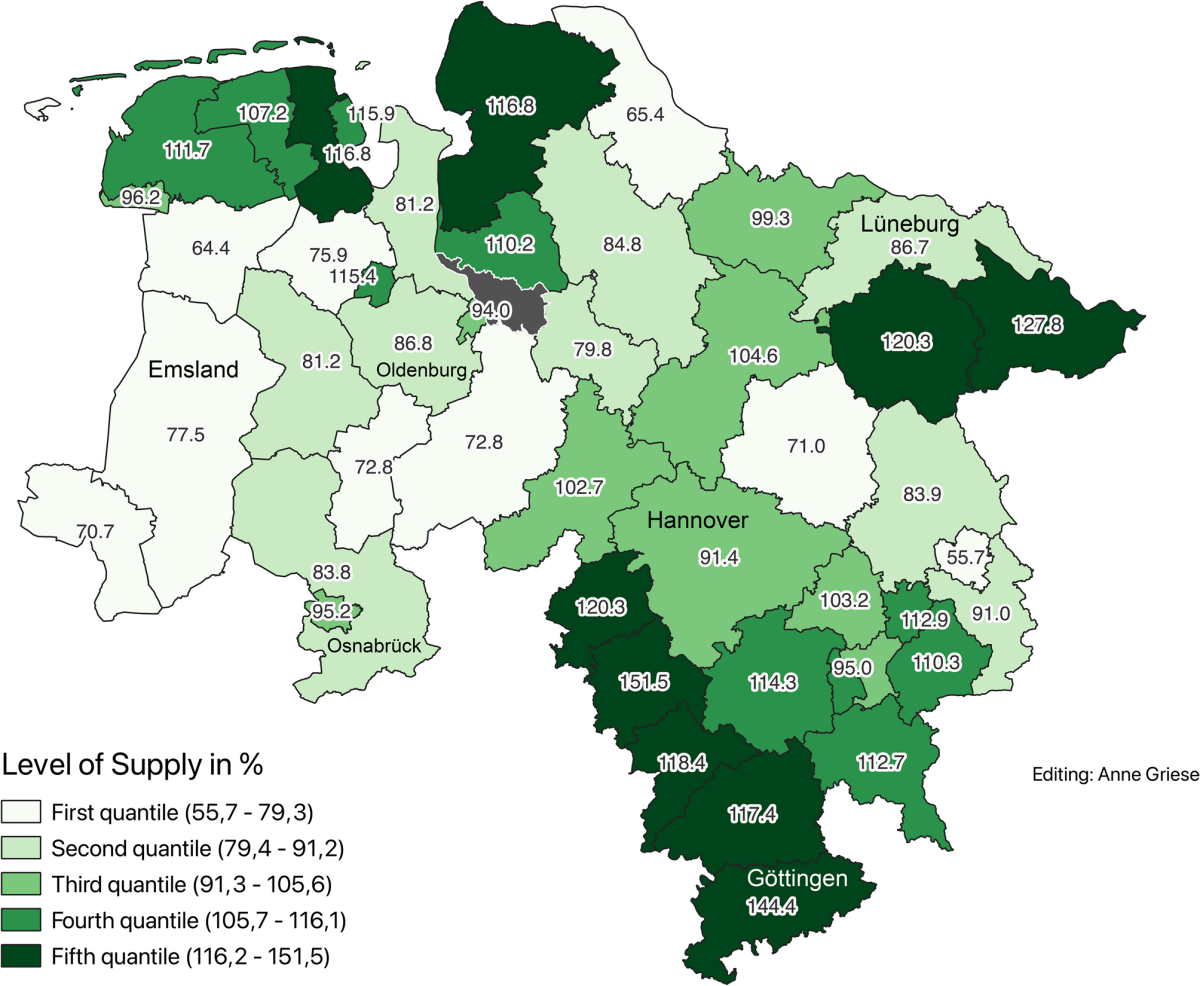
Corrected supply levels by quantiles in 2040 (Data sources: BBSR (2021); Nexiga GmbH (2021); OpenGeoData.NI. (2021))

Using BBSR population forecasts and assuming a constant workforce, future supply levels were calculated. The projections indicate a growing imbalance Figs. 4, 5 and 6:

- 17 districts are expected to be oversupplied ( > 110%)
- 28 districts will remain in the adequate range (50–110%)
- No districts are projected to fall below the 50% threshold

Nevertheless, structural undersupply is likely to persist in several regions with aging populations and low facility densities, notably Wolfsburg.

Projected values for 2040, including the proportion of residents aged 65 and older, total population forecasts, and adjusted supply levels, are listed in Supplementary Table S2.

## Discussion

### Principal findings

This study presents the first application of the physician-based Demand Planning Guideline to physiotherapy in Germany. Using administrative address data from 2021 and population forecasts for 2040, regional supply levels were calculated and adjusted for age structure. Although the overall state average appears balanced, adjusted figures expose clear mismatches at the district level. Several regions face persistent oversupply, while others—especially those with aging populations—show early signs of structural undersupply likely to intensify over time.

### Limitations

Several limitations should be acknowledged. First, the administrative dataset lacks details on practice structure, such as full-time equivalents, treatment volume, or employment status. This may lead to an overestimation of actual care capacity. In addition, in coastal and northern districts some of the apparent oversupply may partly reflect facilities serving seasonal demand from tourism. As the dataset contains only registered business addresses without information on operating hours or service continuity, such seasonal variations cannot be accounted for. Second, demographic adjustment relied solely on age and a national multiplier of 2.1 for individuals aged 65+, without considering morbidity or local utilization rates [10]. Third, the model assumes a static physiotherapy workforce until 2040, although demographic trends and training bottlenecks suggest a likely future decline in workforce capacity [11,19], and potential dynamics such as declining graduate numbers and rising part-time employment may further reduce capacity, whereas recruitment policies or migration could mitigate shortages; however, due to limited longitudinal data, a dynamic projection was not feasible.

### Interpretation

The findings highlight the added value of applying a structured demand planning framework to physiotherapy. While the statewide supply may appear sufficient, regionally disaggregated data reveal mismatches between supply and demand that remain hidden when relying only on aggregated benchmarks. Urban centers with aging populations and limited facility density may face growing care gaps despite being formally classified as adequately supplied.

Recruitment and retention of physiotherapy facilities in rural areas remain challenging. Regional monitoring data for Lower Saxony show persistent outmigration of young adults (18–25 years) from peripheral districts, modest positive balances among families with children in some regions, and positive migration balances for older age groups—indicating rising demand in rural areas where shortages are most likely [20]. In response to these dynamics, the state has already introduced strategies to strengthen rural workforce recruitment in other health professions. These include measures to improve work–family compatibility and regional connectivity, the establishment of multiprofessional regional supply centers, and rural training and incentive schemes such as the Landarztquote for medical students [21,22].

Internationally, structured planning models that include allied health professions have improved service continuity and efficiency. The Netherlands and Norway, for example, employ regional workforce planning models that integrate multiple professions [6,7]. France has introduced direct access to physiotherapy for back pain, reducing GP workload and improving access [23], while the Netherlands has demonstrated improved satisfaction and reduced costs through similar policies [24]. In Sweden and Norway, task-shifting and interprofessional collaboration have helped mitigate regional disparities [9]. Germany currently lacks a comparable framework for allied health professions, even though physiotherapy already accounts for more than 80% of all outpatient therapeutic sessions and the profession is officially classified as a shortage occupation by the Federal Employment Agency, indicating both rising demand and growing workforce shortages [1,10,11].

The ongoing academization of allied health professions may further influence workforce dynamics. Academic training is expected to strengthen professional identity and disciplinary development. In physiotherapy, however, the extent to which academization alone can increase professional attractiveness remains uncertain. Evidence from other countries suggests that positive effects on recruitment and retention are more likely when academic training is accompanied by greater professional autonomy, new areas of responsibility, and appropriate salary adjustments. While this transformation is still ongoing in Germany, its potential impact on workforce distribution remains unclear [25]. Similar uncertainties have been noted in the international literature, which emphasizes that academic reforms must be embedded in broader workforce strategies to address structural shortages effectively [8].

The analytical approach used in this study demonstrates that adapting existing planning frameworks to physiotherapy is both feasible and insightful. By incorporating demographic adjustment, the resulting supply estimates better reflect regional care needs and enable the identification of priority areas for intervention. This is particularly relevant in light of demographic change, as population ageing is expected to shift the geographic distribution and intensity of service demand.

### Generalisability

Although the study is limited to Lower Saxony, the methodological approach is transferable to other federal states in Germany. The use of national demographic forecasts and standardized planning thresholds allows for comparability and replication. Moreover, the framework could be adapted to other outpatient healthcare professions facing similar challenges in distribution and workforce planning. However, regional differences in data availability, healthcare infrastructure, and urban-rural dynamics may influence the extent to which the findings can be directly applied. Still, the results highlight the broader need to expand demand planning beyond physicians to ensure equitable and needs-based service provision across the healthcare system.

## Conclusion

This study highlights significant regional disparities in the distribution of physiotherapy services in Lower Saxony, revealing structural undersupply in districts such as Wolfsburg and notable oversupply in others like Hameln-Pyrmont. These findings illustrate the limitations of current planning mechanisms that exclude allied health professions and rely on aggregated state-level indicators.

Adapting structured planning approaches, such as the Demand Planning Guideline currently applied in medical care, could enable systematic monitoring of regional supply, identification of care gaps, and development of targeted interventions. Such approaches may improve resource allocation, promote interprofessional collaboration, and reduce strain on GPs [9].

To ensure sustainable and equitable care provision, future planning efforts should focus on building centralized provider registries, piloting regionally adaptive models, and strengthening workforce recruitment and retention strategies, particularly considering demographic ageing and increasing service demand.

## Electronic supplementary material

Below is the link to the electronic supplementary material.

Supplementary Material 1

## Data Availability

The datasets generated and/or analyzed during the current study are available from the corresponding author on reasonable request.
