# Supplementary material for: Adapting the German demand planning guideline for physiotherapy: a cross-sectional analysis from Lower Saxony
Source: Res Health Serv Reg. 2025 Dec 12;4:21. doi: 10.1007/s43999-025-00081-1 (PMC12701200; doi:10.1007/s43999-025-00081-1)
Supplement: Supplementary file 1 — Supplementary Material 1 [file 43999_2025_81_MOESM1_ESM.docx]

**Supplements**

**Supplementary Table S1**

Table 1: Regional distribution of physiotherapists and care levels in Lower Saxony (2020).

| City/Region | Population (2020) | Number of Physiotherapists (2020) | Actual Supply Ratio (2020) | Corrected Supply Level in % (2020) |
| --- | --- | --- | --- | --- |
| Hameln-Pyrmont | 146,200 | 50 | 2924.0 | 146.4 |
| Göttingen | 327,500 | 106 | 3089.6 | 142.4 |
| Lüchow-Dannenberg | 47,700 | 14 | 3407.1 | 123.1 |
| Uelzen | 91,900 | 26 | 3534.6 | 121.4 |
| Oldenburg (Oldb), Stadt | 168,500 | 45 | 3744.4 | 121.1 |
| Schaumburg | 156,600 | 44 | 3559.1 | 121.1 |
| Aurich | 189,700 | 50 | 3794.0 | 115.3 |
| Osterholz | 113,300 | 30 | 3776.7 | 115.3 |
| Cuxhaven | 195,800 | 52 | 3765.4 | 113.9 |
| Friesland | 97,200 | 26 | 3738.5 | 113.9 |
| Braunschweig,  Stadt | 249,800 | 63 | 3965.1 | 112.8 |
| Hildesheim | 273,200 | 70 | 3902.9 | 111.7 |
| Northeim | 130,500 | 34 | 3838.2 | 111.4 |
| Holzminden | 69,400 | 18 | 3855.6 | 110.2 |
| Wolfenbüttel | 119,300 | 30 | 3976.7 | 109.5 |
| Wilhelmshaven, Stadt | 75,200 | 19 | 3957.9 | 108.0 |
| Harburg | 254,200 | 62 | 4100.0 | 107.6 |
| Wittmund | 56,300 | 14 | 4021.4 | 107.3 |
| Peine | 133,100 | 32 | 4159.4 | 106.5 |
| Goslar | 135,200 | 34 | 3976.5 | 106.3 |
| Heidekreis | 137,600 | 33 | 4169.7 | 105.2 |
| Nienburg | 119,900 | 28 | 4282.1 | 102.5 |
| Emden, Stadt | 50,600 | 11 | 4600.0 | 97.0 |
| Osnabrück, Stadt | 164,500 | 35 | 4700.0 | 97.0 |
| Delmenhorst,  Stadt | 78,100 | 17 | 4594.1 | 96.3 |
| Lüneburg | 184,900 | 39 | 4741.0 | 94.7 |
| Hannover, Region | 1,160,800 | 246 | 4718.7 | 94.6 |
| Salzgitter, Stadt | 104,000 | 22 | 4727.3 | 92.8 |
| Oldenburg | 130,200 | 27 | 4822.2 | 92.0 |
| Helmstedt | 91,200 | 19 | 4800.0 | 90.7 |
| Osnabrück | 358,300 | 72 | 4976.4 | 89.6 |
| Cloppenburg | 168,700 | 32 | 5271.9 | 87.7 |
| Rotenburg | 162,400 | 32 | 5075.0 | 87.3 |
| Gifhorn | 175,000 | 34 | 5147.1 | 87.1 |
| Verden | 137,100 | 26 | 5273.1 | 83.4 |
| Emsland | 325,000 | 59 | 5508.5 | 82.1 |
| Ammerland | 124,300 | 23 | 5404.3 | 80.9 |
| Wesermarsch | 87,700 | 16 | 5481.3 | 79.5 |
| Vechta | 141,700 | 24 | 5904.2 | 78.6 |
| Diepholz | 215,800 | 37 | 5832.4 | 75.4 |
| Grafschaft | 135,300 | 22 | 6150.0 | 72.6 |
| Celle | 177,800 | 29 | 6131.0 | 71.0 |
| Leer | 169,800 | 26 | 6530.8 | 67.9 |
| Stade | 202,100 | 31 | 6519.4 | 67.9 |
| Wolfsburg, Stadt | 125,200 | 16 | 7825.0 | 56.6 |

**Supplementary Table S2**

Table 2: Projected population demographics and corrected supply levels in 2040 by region.

| City/Region | Population Aged 65+ (2020) in % | Population Aged 65+ (2040) in % | Population Forecast 2040 | Corrected Supply Level in % (2040) |
| --- | --- | --- | --- | --- |
| Hameln-Pyrmont | 26.1 | 31.9 | 134,700 | 151.5 |
| Göttingen | 22.7 | 26.4 | 313,200 | 144.4 |
| Lüchow-Dannenberg | 28.5 | 34.2 | 43,900 | 127.8 |
| Schaumburg | 25.2 | 32.5 | 148,500 | 120.3 |
| Uelzen | 25.8 | 32.8 | 87,600 | 120.3 |
| Holzminden | 26.9 | 33.4 | 61,300 | 118.4 |
| Northeim | 26.1 | 32.7 | 117,500 | 117.4 |
| Cuxhaven | 25.8 | 31.4 | 182,300 | 116.8 |
| Friesland | 26.6 | 32.7 | 90,300 | 116.8 |
| Wilhelmshaven.  Stadt | 26.2 | 29.1 | 68,400 | 115.9 |
| Oldenburg (Oldb),  Stadt | 19.3 | 24.1 | 169,700 | 115.4 |
| Hildesheim | 23.8 | 29.4 | 255,100 | 114.3 |
| Braunschweig,  Stadt | 20.9 | 23.2 | 244,700 | 112.9 |
| Goslar | 27.6 | 32.0 | 123,000 | 112.7 |
| Aurich | 23.5 | 30.4 | 184,800 | 111.7 |
| Wolfenbüttel | 22.0 | 29.4 | 113,300 | 110.3 |
| Osterholz | 24.0 | 30.7 | 112,200 | 110.2 |
| Wittmund | 25.0 | 31.7 | 53,400 | 107.2 |
| Heidekreis | 23.1 | 29.7 | 131,000 | 104.6 |
| Peine | 22.0 | 28.5 | 130,000 | 103.2 |
| Nienburg | 23.1 | 30.6 | 112,400 | 102.7 |
| Harburg | 22.5 | 27.5 | 264,000 | 99.3 |
| Emden, Stadt | 21.1 | 25.2 | 49,300 | 96.2 |
| Osnabrück, Stadt | 18.7 | 22.2 | 162,500 | 95.2 |
| Salzgitter, Stadt | 23.1 | 25.9 | 99,300 | 95.0 |
| Delmenhorst, Stadt | 22.2 | 25.5 | 77,800 | 94.0 |
| Hannover, Region | 21.2 | 24.5 | 1,167,600 | 91.4 |
| Helmstedt | 24.0 | 29.5 | 86,900 | 91.0 |
| Oldenburg | 21.8 | 28.5 | 130,500 | 86.8 |
| Lüneburg | 20.4 | 26.9 | 191,200 | 86.7 |
| Rotenburg | 21.9 | 29.8 | 156,700 | 84.8 |
| Gifhorn | 20.6 | 27.3 | 171,600 | 83.9 |
| Osnabrück | 21.3 | 28.2 | 361,300 | 83.8 |
| Cloppenburg | 17.1 | 24.4 | 171,000 | 81.2 |
| Wesermarsch | 23.9 | 30.7 | 81,200 | 81.2 |
| Verden | 22.8 | 28.5 | 136,700 | 79.8 |
| Emsland | 19.7 | 26.7 | 324,200 | 77.5 |
| Ammerland | 23.6 | 29.8 | 125,800 | 75.9 |
| Diepholz | 22.8 | 29.1 | 212,300 | 72.8 |
| Vechta | 16.8 | 23.4 | 144,300 | 72.8 |
| Celle | 24.0 | 29.2 | 170,300 | 71.0 |
| Grafschaft | 21.1 | 28.2 | 130,800 | 70.7 |
| Stade | 22.0 | 27.9 | 200,000 | 65.4 |
| Leer | 21.8 | 28.6 | 169,200 | 64.4 |
| Wolfsburg, Stadt | 22.0 | 23.4 | 125,700 | 55.7 |
